# Quantal response equilibrium for the Prisoner’s Dilemma game in Markov strategies

**DOI:** 10.1038/s41598-022-08426-3

**Published:** 2022-03-16

**Authors:** T. S. Kozitsina, I. V. Kozitsin, I. S. Menshikov

**Affiliations:** 1 Moscow Institute of Physics and Technology, Institutsky lane 9, Dolgoprudny, Moscow region 141700 Russian Federation; 2 Federal Research Center ″Computer Science and Control″ of the Russian Academy of Sciences, Vavilova street 44/2, Moscow, 119333 Russian Federation; 3 V.A. Trapeznikov Institute of Control Sciences of Russian Academy of Sciences, Profsoyuznaya street 65, Moscow, 117997 Russian Federation

**Keywords:** Human behaviour, Applied mathematics, Computer science

## Abstract

Within the studies of human cooperation, there are gaps that require further investigation. One possible area for growth is developing theoretical concepts which describe high levels of cooperation. In this paper, we present a symmetrical quantal response equilibrium (QRE) in Prisoner’s Dilemma game (PD) constructed in Markov strategies (tolerance to defection and mutual cooperation). To prove the adequacy of the resulting equilibrium, we compare it with the previously found Nash equilibrium in PD in Markov strategies: the QRE converges with the Nash equilibrium that corresponds with the theory. Next, we investigate the properties of QRE in PD in Markov strategies by testing it against experimental data. For low levels of rationality, the found equilibrium manages to describe high cooperation. We derive the levels of rationality under which the intersection between Nash and QRE occurs. Lastly, our experimental data suggest that QRE serves as a dividing line between behavior with low and high cooperation.

## Introduction

There are still gaps in the research of human behavior that require investigation. We know that our way of thinking, actions, and beliefs depend on various internal and external factors. Human behavior includes the important social ability of cooperation as “the act of working together with someone or doing what they ask you”^1^. Perhaps more importantly, cooperation relates to sharing mutual profit, equality, costs, and skills. Throughout the pandemic, we have realized the importance of cooperation for the benefit of society. In fact, human lives have depended on cooperation: wearing a mask, keeping social distance, and practicing patience and generosity with the larger public. Thus, studying people’s ability to cooperate helps us better understand the choices and measures taken during the world pandemic^2^.

Cooperation could be investigated through situations regarded as social dilemmas. In a social dilemma, an individual has a higher profit for defection when others cooperate. However, mutual cooperation leads to better outcomes than mutual defection. For the community, cooperation is preferable to defection; therefore, studying the factors that lead to cooperation is an important step toward understanding people’s behavior in social dilemmas.

Researchers make different arguments for which factors increase cooperation in social dilemmas^3^: using communication^4,5^ or socialization^6–9^, mobility and dynamics^10–12^, connectivity^13–15^, and aspects of an individual’s identity^16,17^. The choice to cooperate is often more of an intuitive act than a meaningful one. It is an emotional, quick, automatic operation that does not involve effort. To support this claim, Rand et al. compared the amount of time that participants in the experiments spent choosing between cooperation and non-cooperation strategies^18^. Their results indicated that quick choice could be one predictor of cooperation. Effects of sociality could also lead to an increased likelihood of cooperation^19^. For example, Capraro et al. introduce the cooperative equilibrium for explaining deviation from the Nash equilibrium, based on the idea that people have some tendency to cooperate by default^20,21^. At the moment, this direction of unselfish behavior research is concentrated around the moral preferences hypothesis which postulates that such a sort of behavior appears because of our “internal standards about what is right or wrong in a given situation” (see Ref.^22^, p. 1).

There are numerous approaches to shifting strategies from individual to social. Which models can explain irrational cooperation in social dilemmas remains a question. Below we list concepts that accept relatively high cooperation levels:

- Quantal response equilibrium^23^.
- Level-k^24^.
- Cognitive hierarchy^25^.
- Quantal level-k^26^.
- Trembling hand perfect equilibrium^27^.
- Proper equilibrium^28^.

Previous studies have demonstrated that social interaction significantly increases the level of cooperation in iterated Prisoner’s Dilemma (PD) games (the most commonly known example of social dilemma), from a 20% cooperation rate prior to socialization to 53% after socialization^9,29–31^. We require a specific approach to model such a high level of cooperative strategy choice. Menshikov et al.^30^ proposed to consider PD in Markov strategies. A symmetric totally mixed Nash equilibrium was found for this game. However, this equilibrium better fits strategies prior to socialization than after. Therefore, we developed a new model that can describe high-cooperation strategies.

## Model

### Prisoner’s Dilemma game (PD)

This work is based on the broadly known PD game. In this game, two participants choose between two strategies: Left or Right for the first participant, and High and Low for the second. The choices are simultaneous and independent from each other. Payoffs correspond to the following payoff matrix (see Table 1), which was employed in the model and experiments. Left and High strategies represent Cooperation, Right and Low represent Defection.

**Table 1:** Payoff matrix in PD game.

Payoff	Left	Right
High	5, 5	0, 10
Low	10, 0	1, 1

### Nash equilibrium for PD

PD has one Nash equilibrium: it is a mutual choice of Defection strategy that gives a payoff of 1 for two players. However, laboratory experiments show that people under some conditions avoid Nash equilibrium^9,30^. For example, individuals under social framing may more frequently choose the Cooperation strategy, behavior that could be considered irrational. For this reason, it would be interesting to discern a theoretical concept underlying this specific behavior.

### Nash equilibrium for PD in Markov strategies

Several researchers^7,9,32,33^ argue that for some subjects, social context led to an increase in cooperative choices of up to 100%. Therefore, the behavior under social context is far from a Nash equilibrium. One way to describe this cooperative behavior is to consider the PD game in Markov strategies.

Consider two participants $i\in \{\mathrm{1,2}\}$. Let us denote the probability of cooperation in round $t$ for the first participant as ${p}_{1}^{c}\left(t\right)$. We describe participants’ behavior by means of the following two quantities: (1) $\gamma$ – *mutual cooperation* (the probability of cooperative choice as the response to the opponent’s cooperative choice on the previous round); (2) $\alpha$ – *tolerance to defection* (the probability of cooperative choice as the response to the opponent’s defection choice on the previous round). Then, we assumed that the decision in round $t$ depends only on the results in round $t-1$. Thus, two variables $\gamma$ and $\alpha$ imply that individuals’ strategies at round $t-1$ completely determine their behavior in round $t$. This model will be referred to as PD in Markov strategies^30,34^. For brevity, we will refer to subsequent γ and α as Markov strategies. The dynamics of participants’ actions can be presented as follows:

1 $$\left\{\begin{array}{c}{p}_{1}^{c}\left(t\right)={\gamma }_{1}{p}_{2}^{c}\left(t-1\right)+{\alpha }_{1}\left(1-{p}_{2}^{c}\left(t-1\right)\right),\\ {p}_{2}^{c}\left(t\right)={\gamma }_{2}{p}_{1}^{c}\left(t-1\right)+{\alpha }_{2}\left(1-{p}_{1}^{c}\left(t-1\right)\right).\end{array}\right.$$

In a stationary state, we have:

2 $$\left\{\begin{array}{c}{p}_{1}^{c}={\gamma }_{1}{p}_{2}^{c}+{\alpha }_{1}\left(1-{p}_{2}^{c}\right),\\ {p}_{2}^{c}={\gamma }_{2}{p}_{1}^{c}+{\alpha }_{2}\left(1-{p}_{1}^{c}\right),\end{array}\right.$$

where ${p}_{1}^{c}$ and ${p}_{2}^{c}$ are stationary probabilities of cooperation.

According to the paper^30^ payoff function for participant 1 takes the following form:

3 $$U\left({p}_{1}^{c},{p}_{2}^{c}\right)=5*{p}_{1}^{c}*{p}_{2}^{c}+0*{p}_{1}^{c}*{(1-p}_{2}^{c})+10*{(1-p}_{1}^{c})*{p}_{2}^{c}+1*(1-{p}_{1}^{c})*(1-{p}_{2}^{c})=-4{p}_{1}^{c}{p}_{2}^{c}-{p}_{1}^{c}+9{p}_{2}^{c}+1.$$

**Figure 1:**
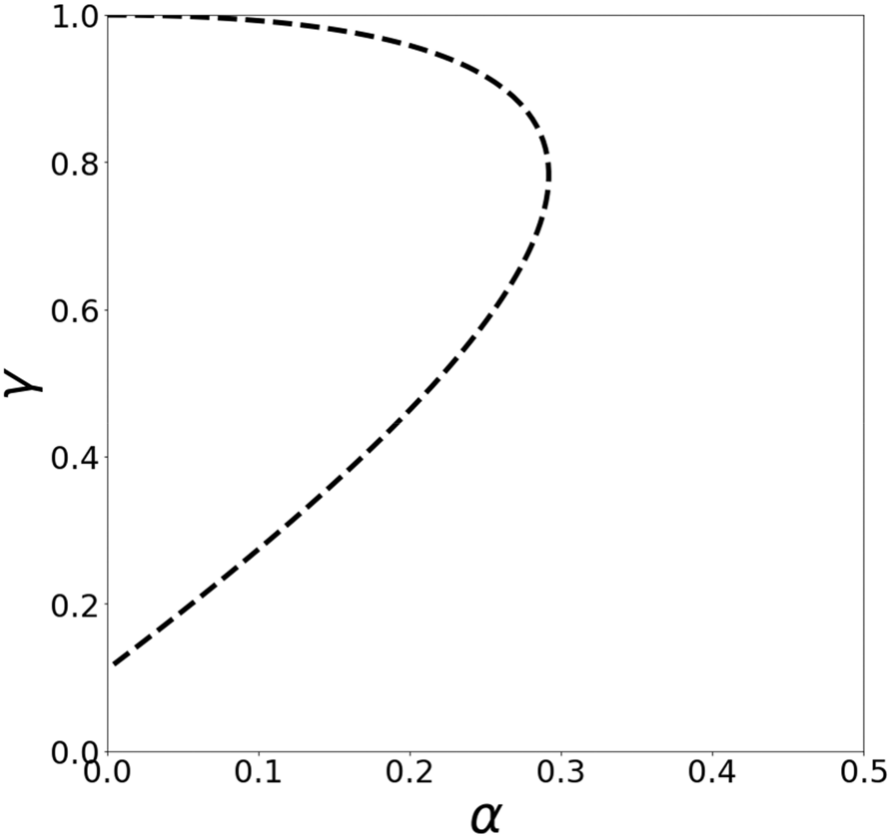
Symmetric totally mixed Nash equilibrium for PD in Markov strategies.

Menshikov et al.^30^ found a symmetric (whereby ${\gamma }_{1}={\gamma }_{2}=\gamma$ and ${\alpha }_{1}={\alpha }_{2}=\alpha$) totally mixed Nash equilibrium for PD in Markov strategies in explicit form. This equilibrium can be represented as the points $\left(\alpha ,\gamma \right)$ that meet the following equation:

4 $$5{\alpha }^{2}+9{\gamma }^{2}-14\alpha \gamma -10\gamma +1=0$$

located in the unit square (see Fig. 1). We will refer to this equilibrium as a Nash equilibrium and this curve as a Nash equilibrium curve.

It is evident from Fig. 1 that curve (4) exists in the area characterized by relatively small values of $\alpha$ (the tolerance to defection does not exceed 0.3). However, experimental results (see “Experimental results” section) reveal that tolerance to defection could exceed 0.5. Also, recent studies indicate that tolerance plays a highly important role in promoting cooperation within a population^35–37^. To reconcile this problem, we derive quantal response equilibrium for PD in Markov strategies in the next section.

### Quantal response equilibrium (QRE)

The QRE model was established to explain participants’ observed behavior in laboratory experiments that differs significantly from the Nash equilibrium^23^. QRE is an internally consistent equilibrium model in the sense that the quantum response functions are based on the distribution of equilibrium probabilities in the choice of strategies of opponents, not simply on arbitrary beliefs that players may have about these probabilities. One feature of this model is that it allows the possibility of player error. QRE requires that expectations must match an equilibrium choice of probabilities. However, in contrast to the classical Nash equilibrium, the definition of QRE assumes that participants strive for the best answer only in the probabilistic sense: the better the answer, the more likely the participant will choose it^38,39^. A comparison of the QRE with experimental data indicated that this approach provided a better fit than the Nash equilibrium^40^. In practice, the QRE is dependent upon employing logistic distribution. The answer ${s}_{i}$ to the mixed strategy ${s}_{-i}$ of the remaining players (the probability of choosing strategy ${s}_{i}$) is expressed through the following formula:

5 $${\sigma }_{i}\left({s}_{i}|{s}_{-i}\right)= \frac{{e}^{\lambda {U}_{i}({s}_{i},{s}_{-i})}}{\sum_{{s}_{i}^{^{\prime}}}{e}^{\lambda {U}_{i}({s}_{i}^{^{\prime}},{s}_{-i})}},$$

where $\lambda$ – is the parameter of a participant’s rationality, and ${U}_{i}({s}_{i},{s}_{-i})$ is the expected gain of participant $i$ when the strategies of other players ${s}_{-i}$ and the strategy ${s}_{i}$ of participant $i$ are given. Therefore, when $\lambda$ → 0 (low rationality), participants chose strategies randomly. When $\lambda$ → ∞ (high rationality), participants chose strategies with no errors (with the highest expected payoff).

The paper^30^ demonstrated that the concept of QRE for a simple PD game works only when the probabilities of cooperative choices do not exceed 50%. Here we present a QRE for PD in Markov strategies. Consider $\left\{{\alpha }_{1},{\gamma }_{1}\right\}$ – Markov strategies of player 1, and $\left\{{\alpha }_{2},{\gamma }_{2}\right\}$ – Markov strategies of player 2, $\left\{{\lambda }_{1},{\lambda }_{2}\right\}$ – players’ rationalities.

From the definition of QRE (5):

6 $$\left\{\begin{array}{c}{\alpha }_{1}= \frac{{e}^{{\lambda }_{1}U({\alpha }_{1},{\gamma }_{1}, {\alpha }_{2},{\gamma }_{2}){\left.\right|}_{{\alpha }_{1}=1}}}{{e}^{{\lambda }_{1}U({\alpha }_{1},{\gamma }_{1}, {\alpha }_{2},{\gamma }_{2}){\left.\right|}_{{\alpha }_{1}=0}}+{e}^{{\lambda }_{1}U({\alpha }_{1},{\gamma }_{1}, {\alpha }_{2},{\gamma }_{2}){\left.\right|}_{{\alpha }_{1}=1}}},\\ {\gamma }_{1}= \frac{{e}^{{\lambda }_{1}U({\alpha }_{1},{\gamma }_{1}, {\alpha }_{2},{\gamma }_{2}){\left.\right|}_{{\gamma }_{1}=1}}}{{e}^{{\lambda }_{1}U({\alpha }_{1},{\gamma }_{1}, {\alpha }_{2},{\gamma }_{2}){\left.\right|}_{{\gamma }_{1}=0}}+{e}^{{\lambda }_{1}U({\alpha }_{1},{\gamma }_{1}, {\alpha }_{2},{\gamma }_{2}){\left.\right|}_{{\gamma }_{1}=1}}},\end{array}\right.$$

where $\left\{{\alpha }_{1},{\gamma }_{1}\right\}\in \left[0;1\right]$ and $\left\{{\alpha }_{2},{\gamma }_{2}\right\}\in \left[0;1\right]$ are unknown, ${\lambda }_{1}\in [0;+\infty )$ is fixed, and $U({\alpha }_{1},{\gamma }_{1}, {\alpha }_{2},{\gamma }_{2})$ is the payoff function. Also, we should mention that ${\alpha }_{1}=0, {\alpha }_{1}=1, {\gamma }_{1}=0, {\gamma }_{1}=1$ are the pure strategies of player 1. To solve system (6), we switched to the symmetrical case by assuming ${\alpha }_{1}= {\alpha }_{2}=\alpha , {\gamma }_{1}={\gamma }_{2}=\gamma ,{\lambda }_{1}={\lambda }_{2}=\lambda$. Next, we determined the equations of payoff function for different cases.

From system (2), we established the following expressions for stationary state probabilities of cooperation:

7 $${p}_{1}^{c}=\frac{{\alpha }_{1}-{\alpha }_{2}({\alpha }_{1}-{\gamma }_{1})}{1-({\alpha }_{1}-{\gamma }_{1})({\alpha }_{2}-{\gamma }_{2})},\quad {p}_{2}^{c}=\frac{{\alpha }_{2}-{\alpha }_{1}({\alpha }_{2}-{\gamma }_{2})}{1-({\alpha }_{1}-{\gamma }_{1})({\alpha }_{2}-{\gamma }_{2})}.$$

Then, we established the profiles of pure strategies for both players when the others’ pure strategies were fixed. To do this, we fixed the profile of strategies ${\alpha }_{2},{\gamma }_{2}, {\gamma }_{1}$ and calculated the probabilities of cooperative choice for pure strategies ${\alpha }_{1}=0$ and ${\alpha }_{1}=1$.

For ${\alpha }_{1}=0$ we expressed:

$$p_{1}^{c} \left. \right|_{{\alpha_{1} = 0}} = \frac{{\alpha_{2} \gamma_{1} }}{{1 + \gamma_{1} \left( {\alpha_{2} - \gamma_{2} } \right)}},\quad p_{2}^{c} \left. \right|_{{\alpha_{1} = 0}} = \frac{{\alpha_{2} }}{{1 + \gamma_{1} \left( {\alpha_{2} - \gamma_{2} } \right)}}.$$

After substituting ${\alpha }_{1}=1$ into (6), we obtained:

$${p}_{1}^{c}{\left.\right|}_{{\alpha }_{1}=1}=\frac{1-{{\alpha }_{2}+\alpha }_{2}{\gamma }_{1}}{1+{(1-\gamma }_{1})({\alpha }_{2}-{\gamma }_{2})},\quad {p}_{2}^{c}{\left.\right|}_{{\alpha }_{1}=1}=\frac{{\gamma }_{2}}{1-({\alpha }_{2}-{\gamma }_{2})(1-{\gamma }_{1})}.$$

Secondly, we fixed the profile of strategies ${\alpha }_{2},{\gamma }_{2}, {\alpha }_{1}$ and obtained the probabilities of cooperative choice for pure strategies ${\gamma }_{1}=0$ and ${\gamma }_{1}=1$.

For $${\gamma }_{1}=0$$:

$${p}_{1}^{c}{\left.\right|}_{{\gamma }_{1}=0}=\frac{{{\alpha }_{1}-\alpha }_{2}{\alpha }_{1}}{1-{\alpha }_{1}({\alpha }_{2}-{\gamma }_{2})},\quad {p}_{2}^{c}{\left.\right|}_{{\gamma }_{1}=0}=\frac{{{\alpha }_{2}-\alpha }_{2}{\alpha }_{1}+{\alpha }_{1}{\gamma }_{2}}{1-{\alpha }_{1}({\alpha }_{2}-{\gamma }_{2})}.$$

When $${\gamma }_{1}=1$$:

$${p}_{1}^{c}{\left.\right|}_{{\gamma }_{1}=1}=\frac{{{\alpha }_{1}+{\alpha }_{2}-\alpha }_{2}{\alpha }_{1}}{1-({\alpha }_{2}-{\gamma }_{2})({\alpha }_{1}-1)},\quad {p}_{2}^{c}{\left.\right|}_{{\gamma }_{1}=1}=\frac{{{\alpha }_{2}-\alpha }_{2}{\alpha }_{1}+{\alpha }_{1}{\gamma }_{2}}{1-({\alpha }_{2}-{\gamma }_{2})({\alpha }_{1}-1)}.$$

In the symmetrical case, we have ${\alpha }_{1}= {\alpha }_{2}=\alpha ,{\gamma }_{1}= {\gamma }_{2}=\gamma$, and ${\lambda }_{1}= {\lambda }_{2}=\lambda$. The probabilities of cooperative choice then took the following forms:

8 $${p}_{1}^{c}{\left.\right|}_{\alpha =0}=\frac{\alpha \gamma }{1+\gamma \left(\alpha -\gamma \right)},\quad { p}_{2}^{c }{\left.\right|}_{\alpha =0}=\frac{\alpha }{1+\gamma \left(\alpha -\gamma \right)},$$

9 $${p}_{1}^{c}{\left.\right|}_{\alpha =1}=\frac{1-\alpha +\alpha \gamma }{1-(1-\gamma )(\alpha -\gamma )},\quad { p}_{2}^{c}{\left.\right|}_{\alpha =1}=\frac{\gamma }{1-(1-\gamma )(\alpha -\gamma )},$$

10 $${p}_{1}^{c}{\left.\right|}_{\gamma =0} =\frac{\alpha -{\alpha }^{2}}{1-\alpha (\alpha -\gamma )},\quad { p}_{2}^{c }{\left.\right|}_{\gamma =0}=\frac{\alpha -{\alpha }^{2}+\alpha \gamma }{1-\alpha (\alpha -\gamma )},$$

11 $${p}_{1}^{c }{\left.\right|}_{\gamma =1}=\frac{2\alpha -{\alpha }^{2}}{1-(\alpha -1)(\alpha -\gamma )},\quad { p}_{2}^{c }{\left.\right|}_{\gamma =1}=\frac{\alpha -{\alpha }^{2}+\alpha \gamma }{1-(\alpha -1)(\alpha -\gamma )}.$$

Note that expressions like $\left(\dots \right){\left.\right|}_{\alpha =0}$ in formulas (8)–(11) are more formal notations than traditional substitution. From this perspective, it is not problematic that expression for ${p}_{1}^{c}{\left.\right|}_{\alpha =0}$ includes $\alpha$. Additionally, despite the symmetrical assumption, formulas (8)–(11) are naturally asymmetric as expressions for ${p}_{1}^{c}$ and ${p}_{2}^{c}$ are different. If the symmetrical assumption was made prior to substituting pure strategies into resulted expressions, a significantly poorer set of (symmetric) stationary state cooperation probabilities would result. Namely, the symmetrical assumption would transform (7) into (for clarity, we utilize capital letters):

$${P}_{1}^{c}={P}_{2}^{c}={P}^{c}=\frac{\alpha -\alpha (\alpha -\gamma )}{1-(\alpha -\gamma )(\alpha -\gamma )}.$$

From there, we derived the following expressions:

12 $$P^{c} |_{{\alpha = 0}} = 0,\;P^{c} |_{{\alpha = 1}} = \frac{1}{2 - \gamma },\;P^{c} |_{{\gamma = 0}} = \frac{\alpha }{1 + \alpha },\;P^{c} |_{{\gamma = 1}} = 1 .$$

It is evident that formula (12) occur only in the extreme cases of strategies (8)–(11). For example, it is true that ${P}^{c}{\left.\right|}_{\alpha =0}={p}_{1}^{c}{\left.\right|}_{\alpha =0}={ p}_{2}^{c }{\left.\right|}_{\alpha =0}$ if we use $\alpha =0$ in (8).

Next, we established the payoff functions using (3) for specific strategies by substituting the found expressions (8)–(11) of probabilities:

13 $$U{\left.\right|}_{\alpha =0}= -4{p}_{1}^{c}{\left.\right|}_{\alpha =0}{ p}_{2}^{c }{\left.\right|}_{\alpha =0}-{p}_{1}^{c}{\left.\right|}_{\alpha =0}+9{ p}_{2}^{c }{\left.\right|}_{\alpha =0}+1 =\frac{-4{\alpha }^{2}\gamma }{{\left(\gamma \left(\alpha -\gamma \right)+1\right)}^{2}}-\frac{\alpha \gamma }{\left(\gamma \left(\alpha -\gamma \right)+1\right)}+\frac{9\alpha }{\left(\gamma \left(\alpha -\gamma \right)+1\right)}+1,$$

14 $$U{\left.\right|}_{\alpha =1}=\frac{-4\gamma (-\alpha \left(1-\gamma \right)+1)}{{(-\left(\alpha -\gamma \right)(1-\gamma )+1)}^{2}}+\frac{9\gamma }{\left(-\left(\alpha -\gamma \right)(1-\gamma )+1\right)}-\frac{\left(-\alpha \left(1-\gamma \right)+1\right)}{\left(-\left(\alpha -\gamma \right)\left(1-\gamma \right)+1\right)}+1,$$

15 $$U{\left.\right|}_{\gamma =0}=\frac{-\left({\alpha }^{2}+\alpha \right)}{\left(-\alpha \left(\alpha -\gamma \right)+1\right)}-\frac{4\left(-{\alpha }^{2}+\alpha \right)\left(-\alpha \left(\alpha -\gamma \right)+\alpha \right)}{{\left(-\alpha \left(\alpha -\gamma \right)+1\right)}^{2}}+\frac{9\left(-\alpha \left(\alpha -\gamma \right)+\alpha \right)}{\left(-\alpha \left(\alpha -\gamma \right)+1\right)}+1,$$

16 $$U{\left.\right|}_{\gamma =1}=\frac{-(\alpha (\alpha -1)+\alpha )}{(-(\alpha -1)\left(\alpha -\gamma \right)+1)}-\frac{4\left(-\alpha \left(\alpha -1\right)+\alpha \right)\left(-\alpha \left(\alpha -\gamma \right)+\alpha \right)}{{\left(-\left(\alpha -1\right)\left(\alpha -\gamma \right)+1\right)}^{2}}+\frac{9\left(-\alpha \left(\alpha -\gamma \right)+\alpha \right)}{\left(-\left(\alpha -1\right)\left(\alpha -\gamma \right)+1\right)}+1.$$

Finally, we used the found expressions (13)–(16) of the payoff functions in system (6) that gave:

17 $$\left\{\begin{array}{c}\alpha = \frac{{e}^{\lambda U{\left.\right|}_{\alpha =1}}}{{e}^{\lambda U{\left.\right|}_{\alpha =0}}+{e}^{\lambda U{\left.\right|}_{\alpha =1}}},\\ \gamma = \frac{{e}^{\lambda U{\left.\right|}_{\gamma =1}}}{{e}^{\lambda U{\left.\right|}_{\gamma =0}}+{e}^{\lambda U{\left.\right|}_{\gamma =1}}},\end{array}\right.$$

where $\alpha \in \left[0;1\right]$ and $\gamma \in \left[0;1\right]$ are unknown, $\lambda \in [0;+\infty )$ is fixed, and $U$ is the payoff function. Note that different values of the rationality could lead to different profiles of strategies.

We propose to solve system (17) numerically, reducing it to finding (as feasibly as possible) the optimal solution for the following optimization problem:

18 $$\left\{\begin{array}{c}\underset{\alpha ,\gamma }{\mathrm{min}}{(\frac{{e}^{\lambda U{\left.\right|}_{\alpha =1}}}{{e}^{\lambda U{\left.\right|}_{\alpha =0}}+{e}^{\lambda U{\left.\right|}_{\alpha =1}}}-\alpha )}^{2}+{(\frac{{e}^{\lambda U{\left.\right|}_{\gamma =1}}}{{e}^{\lambda U{\left.\right|}_{\gamma =0}}+{e}^{\lambda U{\left.\right|}_{\gamma =1}}}-\gamma )}^{2},\\ \alpha \in \left[0;1\right],\\ \gamma \in \left[0;1\right].\end{array}\right.$$

To solve optimization problem (18)$,$ we used the Python package *minimize* from scipy.optimize^41^. We solved the problem (18) for different values of $\lambda$ shifting from $\lambda =0$ to $\lambda =200$ with the step of 0.01. As the result, the solution of optimization problem (18) gave us the set of points ($\alpha , \gamma$) for values of $\lambda \in [0;200]$ – sought-for QRE for PD in Markov strategies. To prove the adequacy of the optimization method, we investigated the behavior of the objective function:

19 $$f={\left(\frac{{e}^{\lambda U{\left.\right|}_{\alpha =1}}}{{e}^{\lambda U{\left.\right|}_{\alpha =0}}+{e}^{\lambda U{\left.\right|}_{\alpha =1}}}-\alpha \right)}^{2}+{\left(\frac{{e}^{\lambda U{\left.\right|}_{\gamma =1}}}{{e}^{\lambda U{\left.\right|}_{\gamma =0}}+{e}^{\lambda U{\left.\right|}_{\gamma =1}}}-\gamma \right)}^{2}.$$

**Figure 2:**
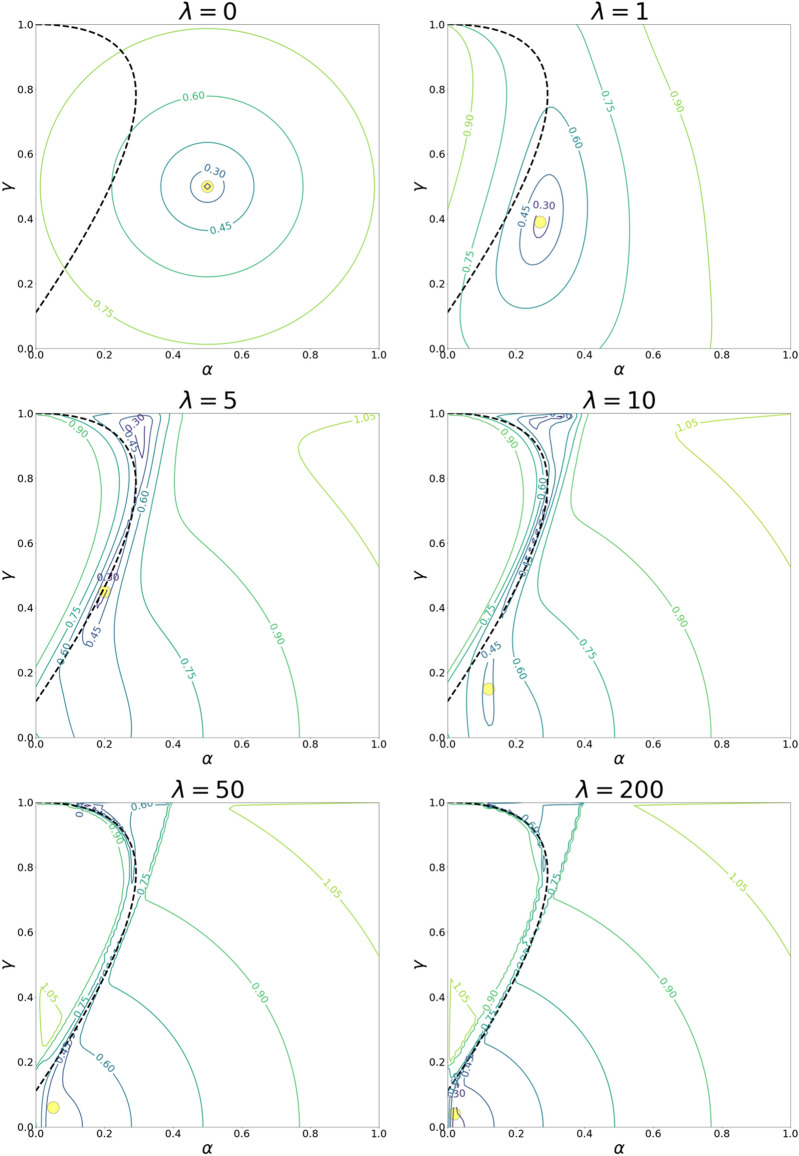
The panels plot contour lines of objective function (19) for different values of rationality (the objective function is shown to the power of 1/5 for demonstrative purposes). The dashed line represents the Nash equilibrium for PD in Markov strategies. The yellow circles symbolize solutions to corresponding optimization problem (18).

In Fig. 2, we illustrate the contour lines of objective function (19) for different values of rationality $\lambda$ with the solutions of optimization problem (18) for the same value of rationality (yellow points) and with the Nash equilibrium curve. We noticed that for small values of $\lambda <\sim 4$, objective function (19) has a unique local minimum. Additionally, the optimization method found the solution within these areas of the local minimum. However, for $\lambda >\sim 4,$ objective function (19) contained more than one local minimum and most of these local minimums were located within the Nash equilibrium curve. For $\lambda \in (\sim 4,\sim 7)$, the optimization method found the global minimum inside one of the local minimums. For $\lambda >\sim 7,$ the results of the optimization method started to shift to the area within the standard Nash equilibrium $\alpha =0,\gamma =0$ (defection/defection) and finally reached a point outside the Nash equilibrium curve in local minimums of objective function (19). This result corresponds to the theory, as the Nash equilibrium describes the behavior of fully rational participants^23^. Therefore, we can conclude that the chosen optimization method worked sufficiently.

**Figure 3:**
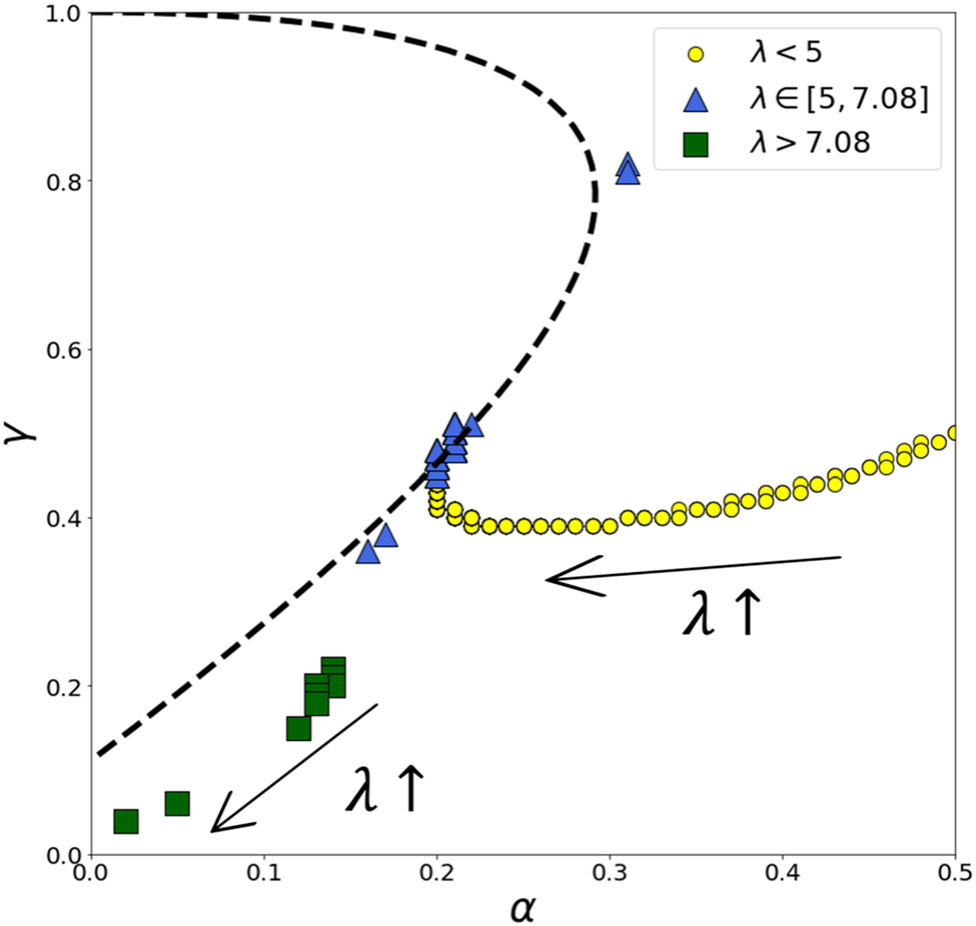
QRE for PD in Markov using solving optimization problem (18) for different values of rationality. The arrows indicate the directional growth of rationality. The resulting QRE curve contains three branches that correspond to various ranges of rationality. The dashed line represents the Nash equilibrium.

In Fig. 3, we plot the arrangement of the obtained QRE for PD in Markov strategies (the solution of (18)) and the Nash equilibrium curve. The QRE for PD in Markov strategies forms an almost near smooth curve in the range of small $\lambda$ (approximately less than 5). For these values of rationality, objective function (19) consistently showed a unique global minimum that was perfectly caught by the solver. In the middle range of $\lambda$ (approximately located at the interval $\left[\mathrm{5,7.08}\right]$), the solution of optimization problem (18) approaches the Nash equilibrium curve that corresponds to the theory^23^. Nonetheless, at these levels of rationality, the QRE for PD in Markov strategies curve (further, QRE curve) loses its smoothness and solutions “leapfrog” on the Nash equilibrium curve (see the blue triangles on Fig. 3). This could be the result of the optimization method weakness (in Fig. 2, we demonstrate that when $\lambda >\sim 5$ objective function (19) has many local minimums). However, the exact “first” intersection between the QRE curve and the Nash equilibrium curve (which is located near $\alpha \approx 0.2,\gamma \approx 0.5$ and is derived under $\lambda \approx 5$) fits the experimental data best when compared to other intersections (see “Experimental results” section). For large values of the rationality ($\lambda >7.08$), solutions of (18) converge to the point $\alpha =0,\gamma =0$ which does not belong to the Nash equilibrium curve. Instead, it marks the strategies’ profiles of the standard Nash equilibrium (defection/defection).

## Experimental results

In this section, we evaluate the equilibrium found against the data from laboratory experiments which were presented in several publications^9,29,30^. The primary goal of these experiments was to identify the effect of socialization on the level of cooperation choice in the PD game.

The full description of the experiments (N = 14) can be found in Supplementary 1. The following is a schematic representation of the experimental design:

1. The sample for one experiment included 12 recruited participants (all strangers).
2. Participants played iterated PD (Table 1) in a mixed-gender group of 12 participants for 11–22 rounds.
3. Socialization occurred across unacquainted group members, who were divided into two groups of 6 participants.
4. Participants played iterated PD (Table 1) in the newly formed groups for 15–20 rounds.
5. Participants were compensated for the experiment.

The study procedures involving human participants were approved by the Skolkovo Institute of Science and Technology (Skoltech) Human Subjects Committee. Written informed consents were obtained from participants. All methods were performed in accordance with the relevant guidelines and regulations.

In Supplementary Table S1 (Supplementary 2), we present aggregated results of the 14 experiments (168 participants). We find that the choice of cooperation is higher after socialization (58%) rather than before (22%). We assume that socialization compensates for the irrationality of these choices. This implies that despite the expectation that the payoff of defection is higher than that of cooperation, the utility of sociality is higher than the probable losses of the cooperation choice. In comparing theoretical results with the experimental data, we found for every part of the experiments probabilities of mutual cooperation ($\gamma$) and tolerance to defection ($\alpha$) in all parts of the experiments. Thus, we analyzed the aggregating strategies by participants that represent the most popular strategies in the observed part of the experiment. Due to the experimental design, we aggregated strategies by all 12 participants for every experiment before socialization. We aggregated strategies by two socialized groups of six participants for every experiment after socialization (see Supplementary Table S1, Supplementary 2).

**Figure 4:**
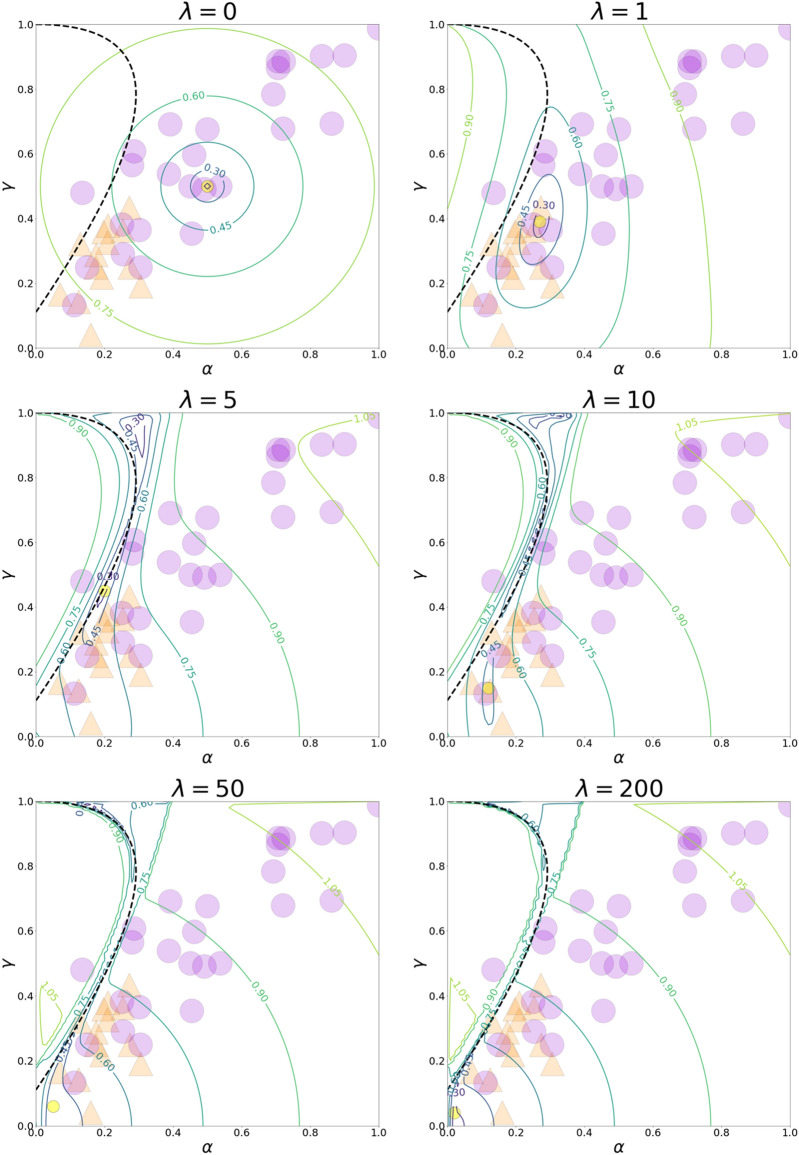
The panels plot contour lines of objective function (19) (the objective function is shown to the power of 1/5 for demonstrative purposes) for different values of rationality. The dashed line represents the Nash equilibrium. The yellow circles symbolize solutions to the corresponding optimization problems (QRE for PD in Markov strategies). The orange triangles indicate participants’ strategies before socialization, while violet circles represent participants’ strategies after socialization.

**Figure 5:**
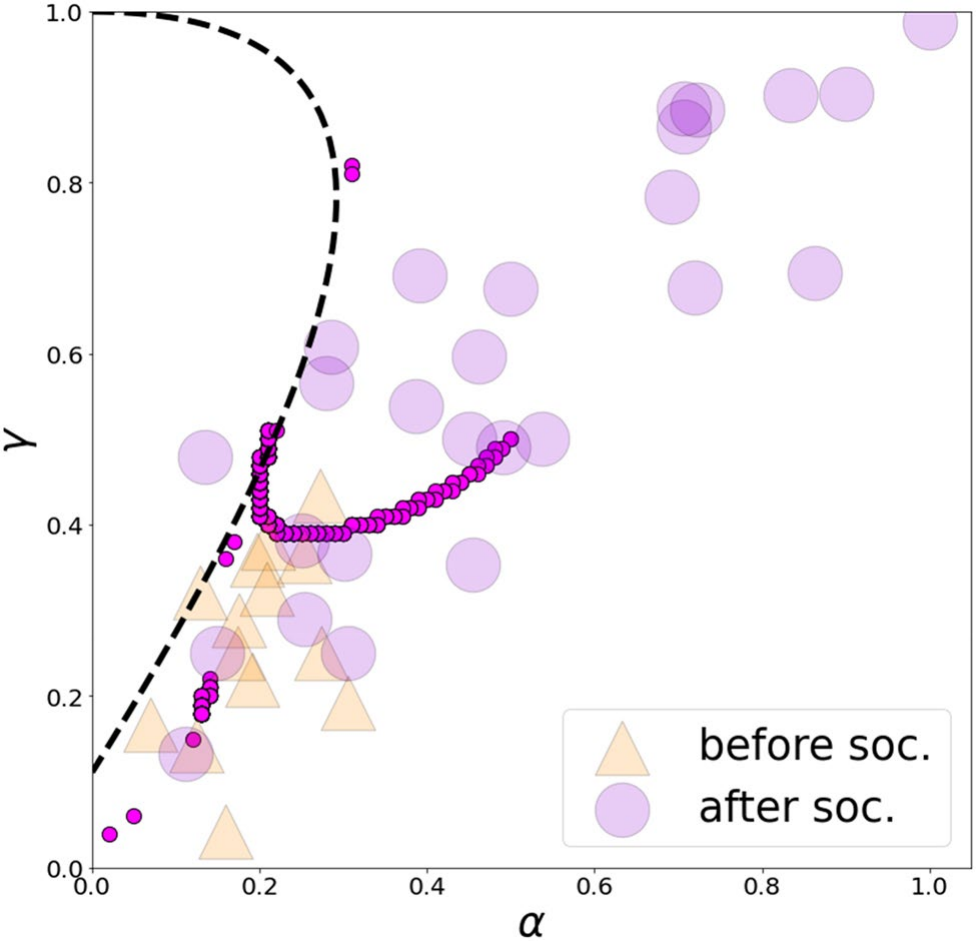
The dashed line represents the Nash equilibrium. The pink circles represent the QRE curve. The orange triangles signify participants’ strategies before socialization, while the violet circles signify participants’ strategies after socialization. The QRE for PD in Markov strategies points for $\lambda <\sim 5$ serve as a natural border between points before and after socialization.

We first analyzed how experimental points corresponded to values of objective function (19) under different levels of rationality (see Fig. 4). We observed that most participants’ strategies could be approximated by the minima of the objective function after selecting the appropriate level of rationality. More precisely, we recognized that the behavior of individuals with a high level of cooperation (more than 50%) could be modeled by selecting low rationality rates (which was one of our objectives), whereas low-cooperative participants were well-approximated by high values of rationality. Unfortunately, participants located in the upper right zone of the phase plane are still unexplained. Notably, among all the Nash equilibrium, the best equilibrium that fits the experimental data was found on the initial intersection between the QRE curve and the Nash equilibrium curve (at the rationality level $\lambda \approx 5$). The QRE for PD in Markov strategies for $\lambda <\sim 5$ divided most of the strategies before and after socialization (see Fig. 5), resembling phase boundary.

## Discussion and conclusion

In an era that highlights the importance of every individual’s choice while promoting living for oneself, it is crucial to consider cooperation as an effective mechanism to promote the overall well-being of society. In this paper, we present a theoretical concept that sufficiently works well enough with high cooperation levels that were previously obtained in the laboratory experiments^9,30^, that examining the influence of socialization on strategy choices in the PD game. In this paper, we present a symmetrical QRE for PD in Markov strategies (further, QRE-PD-M) and compared it with the symmetrical Nash for PD in Markov strategies^30^ and experimental results^9^. Under the PD game in Markov strategies, we specify the following: a) instead of pure PD strategies (cooperation or defection), we employ mutual cooperation (the probability of a cooperative choice in response to an opponent’s choice in the previous round), and tolerance to defection (the probability of a cooperative choice as the response to an opponent’s defection choice in the previous round); and b) the choice of strategy in the current period depends solely on the strategies from the previous period.

When we matched the QRE-PD-M and the experimental data, we observed that the QRE-PD-M with the low rationality rates described high levels of cooperation after socialization. Conversely, the QRE-PD-M with the high values of rationality approximates low-cooperative results before socialization. We also found how the QRE-PD-M completes the Nash equilibrium. The intersection between the equilibrium curves under the low parameter of rationality ($\lambda \approx 5$) gives the unique selection of Nash equilibrium, most closely fitting the experimental data the closest (compared to other Nash equilibria for PD in Markov strategies). Additionally, the QRE-PD-M curve (for the parameter of rationality $\lambda <\sim 5$) serves somewhat as a phase boundary for the experimental data before and after socialization; most of the points for before socialization lie below the QRE-PD-M while most of the points for after socialization lie above. However, this result may be coincidental.

Our study is not without limitations. They are as follows:

1. We found and described only the symmetrical case of the QRE-PD-M. Symmetricity allows us to avoid some mathematical difficulties. However, by doing so, we likely lose some equilibria.
2. The optimization problem was solved using numerical methods that come with possible noise in the solutions.
3. We compared the theoretical results against the experiments that involved only one socialization strategy. Further, these experiments were performed within the same socio-demographic and cultural context.

In this paper, our initial motivation was to explain the high levels of cooperation observed in empirics via the concept of QRE. In a nutshell, we obtained that the found QRE-PD-M curve fits the data well. On the one hand, it means that we succeed in our purpose. On the other hand, one could wonder that our approach is rather meaningless because QRE is drawn upon the idea that individuals may “make errors,” and thus, our explanation of high cooperation via the QRE approach could imply that cooperation is simply “an error”.

To reconcile this problem, we put forward the following hypothesis (which should be carefully tested in future studies!) whereby we attempt to bring together our findings and the modern notion of what is a rational sort of behavior. First, we propose that the segment of the QRE-PD-M curve that is characterized by small values of $\lambda$ ($\lambda <\sim 5$; hereafter, the small-rationality segment) serves as a natural indicator of a participant’s state. This state could be individual (leading to selfish behavior), or social (in the social state, individuals should act unselfishly). Further, we suggest that the difference between these two states lies in an individual’s utility function. According to the moral preferences hypothesis, the utility function includes the “moral component” that describes our “internal standards about what is right or wrong in a given situation”^22^. We suggest that in the individual state, the moral component is not well pronounced and doesn’t compensate for the risk of cooperative behavior according to our internal moral standards (e.g., behavior in a group of unfamiliar people). Thus, the rational (from the classical view) sort of behavior should happen (corresponding strategies are located beneath the small-rationality segment). In the social state, this component comes with more effect to the utility function because of our need to belong to the social group^42^ (and behave in a more empathetic and sensitive manner towards the group members). Then, people in the social state prefer to cooperate rather than defect (strategies above the small-rationality segment).

In other words, if individuals’ strategies fall beneath the small-rationality segment, then we conclude that the people are in the individual state. If strategies are above the small-rationality segment, then we observe the social state. In the case the strategies are located near the small-rationality segment, then we hypothesize that the corresponding individuals are in “an intermediate regime.” In our empirical context, the changes in participant behavior were caused by the socialization procedure that made the inter-group relations more solid and thus increased the effect of the moral component. Because of this issue, participants’ strategies had a tendency to move from the individual to the social state.

However, questions remain: (1) why does the small-rationality segment play this “dividing” role and (2) how should we interpret strategies that are located near the small-rationality segment? We propose the following answer: one could think about the small-rationality segment as a zone where individuals experience a phase transition between the individual and social states. In this intermediate regime, the participants’ behavior becomes rather “unpredictable” (people face considerable uncertainties about choosing between selfish and unselfish behavior) and thus may be described meaningfully via the concept of QRE. Depending on individuals’ personal characteristics and the socio-demographic and cultural contexts, and, importantly, on the effectiveness of socialization, individuals may find themselves into individual, intermediate, or social states both before and after socialization. Figure 5 may be considered as evidence to support this statement.

A promising avenue for future studies would be to test our hypothesis. We believe that it could be done in two directions: (1) theoretical—by deriving corresponding models that consider a utility function from which the small-rationality segment and the corresponding unpredictable regime should come as a special case that marks the phase transition between individual and social states and (2) empirical—by conducting more experiments involving different socio-demographic and cultural contexts as well as various socialization strategies. Apart from socialization^9,43^, the phase shift could be triggered by nudging personal norms, turning on morality, and other methods presented by Capraro and Perc^22^. These experiments may reveal whether the small-rationality segment serves as a boundary between the individual and social states.

If the theory is found to be true, then this segment can be considered as a universal (and, importantly, theoretically motivated) indicator of whether the social relationships in a group of individuals are solid enough. This question is extremely important because solid relationships ensure that the group members are open to cooperative actions and the group can solve complex tasks that require high levels of collaboration. From this perspective, our results could be implemented in team-building activities. For example, one could use the PD game to analyze how participants act before and after implementing team-building tasks. If the tasks were implemented successfully, then it is reasonable to expect that the participants chose strategies above the small-rationality segment afterwards. Further, this methodology could also be used to compare different team-building strategies.

## Supplementary Information

Supplementary Information.
