# Supplementary material for: Quantal response equilibrium for the Prisoner’s Dilemma game in Markov strategies
Source: Sci Rep. 2022 Mar 16;12:4482. doi: 10.1038/s41598-022-08426-3 (PMC8927616; doi:10.1038/s41598-022-08426-3)
Supplement: Supplementary file 1 — Supplementary Information. [file 41598_2022_8426_MOESM1_ESM.docx]

## Quantal response equilibrium for the Prisoner’s Dilemma game in Markov strategies

T.S. Kozitsina ^a,b*^, I.V. Kozitsin ^c,a^, I.S. Menshikov ^a,b^

^a^ Moscow Institute of Physics and Technology (National Research University), Moscow, Russian Federation

^b^ Federal Research Center Computer Science and Control, Russian Academy of Sciences, Moscow, Russian Federation

^c^ V.A. Trapeznikov Institute of Control Sciences, Russian Academy of Sciences, Moscow, Russia

* Corresponding author

E-mail: [tatyana.babkina@phystech.edu](mailto:tatyana.babkina@phystech.edu)

**Supplementary 1**

Participants were recruited from the Moscow Institute of Physics and Technology in Moscow. A total of 168 individuals (72 females) participated in 14 experiments. We recruited participants through posting advertisements on the social networking site VKontakte. For every experiment, we only selected participants who were unacquainted with each other. Because our participants were students, we collected demographic information, such as academic major, group, and year of study. All participants were provided with written and verbal instructions related to the experiment. Experimenters notified participants that all points won in the games would be converted to real money (the average win rate was approximately equal to the cost of a full lunch in a cafe). Experimental data are available from the authors. The experimental design and the results are also presented in the following papers^1,2,3^.

**Game*.*** The study employed the Prisoner’s Dilemma Game (PD).

**Iterated Prisoner’s Dilemma Game.** Two individuals anonymously participated in each round of the game. They both had two strategies: cooperation or defection (Table 1). Participants were divided into pairs randomly each period of the game.

**Experimental design.** The experimental procedure consisted of three stages. To execute the game, a specialized tool “z-Tree” developed at the University of Zurich for designing and performing experiments in a group of experimental economics was used^4^.

**Stage 1: Anonymous playing phase.** Participants played the Prisoner’s Dilemma Game for eleven to twenty-two game rounds. Participants did not know how many rounds they would play. In each round, participants were randomly divided into pairs and made choices simultaneously and independently of each other. In each round, participants were re-paired randomly, and participants were unaware of who they were playing against. After each of the periods, each participant observed their own and their opponent’s results on a screen.

Points earned at this stage were added to the total win and converted into real money at the end of the game.

**Stage 2: Socialization phase.** In this phase, participants engaged in social interaction, which consisted of familiarization, communication, and division into groups. The participants memorized each other’s names by playing "snowball"^1,2^. According to the game, players were seated in a circle, and the first person said his/her name and a personal quality that started with the same letter as the name. Second, the next participant repeated the name along with the quality of the first participant and gave his/her name and quality. This process was repeated with each participant until the last person, who was due to repeat all the names and personal qualities. Then, in a different order, the participants shared personal information, such as their hometown, academic major, hobbies, and interests. Following that, two captains were volunteer selected themselves from among the participants. The captains remained indoors while the other participants left the room. Then, in random order, they entered the room one by one. Every participant who entered the room chose a captain whose group he/she wanted to join. Consequently, two groups of 6 people were formed. In the end, each group of 6 people was tasked to find 5 common characteristics (i.e., 5 characteristics that united them) and choose a name for their group.

**Stage 3. Socialized phase.** The participants played the Prisoner's Dilemma Game. However, unlike the first stage, the participants interacted only in the groups of 6 previously composed during the socialization phase of the experiment. For each round, the participants were randomly divided into pairs. They were informed that they were interacting with a member of their "own" group, but they did not know who exactly that person was. After each of the periods, each participant observed their own and their opponent’s results on a screen. Both the games consisted of 15-20 rounds. The group names, chosen by participants at the socialization stage. appeared on monitors in the Prisoner's Dilemma Game.

Points were added to those obtained at the first stage. As a result, the final prize was generated and could be converted into a cash reward to compensate participants.

1. Babkina, T. *et al.* Choice of the group increases intra-cooperation. in *CEUR Workshop Proceedings* **1627**, (2016).

2. Peshkovskaya, A., Babkina, T. & Myagkov, M. Social context reveals gender differences in cooperative behavior. *J. Bioeconomics* (2018). doi:10.1007/s10818-018-9271-5

3. Menshikov, I. S., Shklover, A. V., Babkina, T. S. & Myagkov, M. G. From rationality to cooperativeness: The totally mixed Nash equilibrium in Markov strategies in the iterated Prisoner’s Dilemma. *PLoS One* **12**, (2017).

4. Fischbacher, U. z-Tree: Zurich toolbox for ready-made economic experiments. *Experimental economics* **10(2),** 171-178 (2007).

**Supplementary 2**

Supplementary Table S1. Experimental results aggregated by experiments.

| **Number of the experiment** | **% of cooperation before socialization** | **alpha before socialization** | **gamma before socialization** | **% of cooperation after socialization** | | **alpha after socialization** | | **gamma after socialization** | |
| --- | --- | --- | --- | --- | --- | --- | --- | --- | --- |
|  |  |  |  | group 1 | group 2 | group 1 | group 2 | group 1 | group 2 |
| Exp_1 | 18.89% | 0.19 | 0.22 | 45% | 28% | 0.39 | 0.30 | 0.54 | 0.25 |
| Exp_2 | 13.89% | 0.16 | 0.04 | 30% | 48% | 0.25 | 0.45 | 0.38 | 0.50 |
| Exp_3 | 17.22% | 0.13 | 0.32 | 34% | 85% | 0.30 | 0.71 | 0.37 | 0.87 |
| Exp_4 | 29.17% | 0.25 | 0.36 | 100% | 77% | - | 0.69 | 1.00 | 0.78 |
| Exp_5 | 25.38% | 0.20 | 0.36 | 54% | 41% | 0.46 | 0.28 | 0.60 | 0.57 |
| Exp_6 | 14.02% | 0.12 | 0.14 | 90% | 18% | 0.83 | 0.15 | 0.90 | 0.25 |
| Exp_7 | 26.52% | 0.21 | 0.38 | 84% | 86% | 0.72 | 0.71 | 0.89 | 0.89 |
| Exp_8 | 9.47% | 0.07 | 0.17 | 13% | 53% | 0.11 | 0.54 | 0.13 | 0.50 |
| Exp_9 | 20.56% | 0.17 | 0.25 | 28% | 58% | 0.25 | 0.39 | 0.29 | 0.69 |
| Exp_10 | 28.33% | 0.31 | 0.19 | 22% | 49% | 0.13 | 0.49 | 0.48 | 0.49 |
| Exp_11 | 25.56% | 0.21 | 0.33 | 63% | 100% | 0.50 | - | 0.68 | 1.00 |
| Exp_12 | 20.45% | 0.18 | 0.29 | 45% | 90% | 0.29 | 0.90 | 0.61 | 0.90 |
| Exp_13 | 33.33% | 0.27 | 0.43 | 74% | 41% | 0.86 | 0.45 | 0.69 | 0.35 |
| Exp_14 | 28.79% | 0.27 | 0.25 | 99% | 70% | 1.00 | 0.72 | 0.99 | 0.68 |
| **Mean:** | **22.25%** | **0.20** | **0.27** | **56%** | **60%** | **0.47** | **0.52** | **0.61** | **0.62** |
